# Polysaccharide-Based Membranes in Food Packaging Applications

**DOI:** 10.3390/membranes6020022

**Published:** 2016-04-13

**Authors:** Ana R. V. Ferreira, Vítor D. Alves, Isabel M. Coelhoso

**Affiliations:** 1 LAQV-REQUIMTE, Departamento de Química, Faculdade de Ciências e Tecnologia, Universidade NOVA de Lisboa, Caparica 2829-516, Portugal; imrc@fct.unl.pt; 2 LEAF—Linking Landscape, Environment, Agriculture and Food, Instituto Superior de Agronomia, Universidade de Lisboa, Tapada da Ajuda, Lisboa 1349-017, Portugal; vitoralves@isa.ulisboa.pt

**Keywords:** polysaccharides, microbial polysaccharides, packaging, biopolymer-derived membranes

## Abstract

Plastic packaging is essential nowadays. However, the huge environmental problem caused by landfill disposal of non-biodegradable polymers in the end of life has to be minimized and preferentially eliminated. The solution may rely on the use of biopolymers, in particular polysaccharides. These macromolecules with film-forming properties are able to produce attracting biodegradable materials, possibly applicable in food packaging. Despite all advantages of using polysaccharides obtained from different sources, some drawbacks, mostly related to their low resistance to water, mechanical performance and price, have hindered their wider use and commercialization. Nevertheless, with increasing attention and research on this field, it has been possible to trace some strategies to overcome the problems and recognize solutions. This review summarizes some of the most used polysaccharides in food packaging applications.

## 1. Introduction

Food packaging is essential for products containment, protection, preservation, convenience, to provide information about the product, brand communication, among others. This work is focused on primary packaging that is in direct contact with food, such as bottles, trays or bags [1]. This type of packaging is the most familiar to consumers and is defined as “a sales unit to the final user or consumer at the point of purchase” [2].

It should prevent or reduce products damage and food spoilage, reduce or eliminate the risk of adulteration and present food in a hygienic and aesthetically attractive way. Essentially, packaging strategies result from the combination of food science, processing and preservation, once they must extend the shelf life of food products reducing the wastage [1,3].

Plastic packaging represents almost 40% of the European plastics market and is essential for processing, storing, transporting, protecting and preserving food [4,5]. In fact, over 50% of all European goods are packaged in plastics, and this commercial success is due to a combination of properties such as flexibility, strength, lightness, stability, impermeability and ease of sterilization. These characteristics make them ideal materials for almost every commercial and industrial consumer [6]. According to the last report of Plastics Europe—Association of plastics manufacturers (2015) [5], the plastics production has grown globally and is stable in Europe (57 Mtonne per year). This success of plastics comes from the fact that many of them can be molded, extruded, cast and blown in different shapes, films/membranes or foams [5,7].

Polyethylene (PE), polypropylene (PP) and polyethylene terephthalate (PET) are the most used in the packaging sector [8], but polyvinyl chloride (PVC) and polystyrene (PS) are also easily found in food packaging due to their biological resistance and excellent water barrier properties [9].

The real success of plastics in food packaging industry is achieved with combination of all referred characteristics (in particular lightness) and their use to help keep food fresh and free of contamination [8]. The extended shelf life of food products has been reported for different foods with simple packaging; for example, unwrapped cucumber loses moisture and becomes dull and unsaleable within three days, but 1.5 g of plastic wrapping can keep a cucumber fresh for 14 days. Some more complex plastic packaging can extend (three times) the shelf-life of specific products, due to unique properties, such as resealable portioned packs, anti-microbial agents, humidity control systems and modified atmosphere packaging solutions. Furthermore, it is expected that in the near future more innovations will be available in large scale and at low price, such as absorbers and emitters of naturally occurring gaseous substances to prolong products shelf life, biosensors that detect bacteria or printable RFID (radio-frequency identification) tags to inform about integrity of the products [4,10].

Plastic membranes are usually produced by extrusion, co-extrusion, casting, extrusion coating, extrusion lamination and metallization. These processes have advantages and disadvantages depending on the polymer used and the thickness required, usually less than 250 μm [9].

However, the crucial problem of using plastics for packaging is the post-consumer waste, once packaging is by far the largest contributor (63%) of plastic waste [11]. In addition, some materials are difficult to reuse and it is estimated that less than 14% of plastic packaging materials are recyclable [12]. According to the report Plastics—The facts 2014/2015 [5], despite recycling and energy recovery solutions have increased since 2006, the landfill disposal remains the largest solution for plastic in the end-of-life, making 9.5 M tonne of plastic waste (38% of the total) in 2012, for EU27 + Switzerland and Norway. Because most materials used are non-biodegradable, which remain in the environment for long periods of time, they remain threats to human health as well as to the environment [5].

The other used solution, incineration for energy recovery, has a severe environmental impact (as for most solid wastes or fuels), which can include some airborne particulates and greenhouse gas emissions [11].

According to these facts and knowing that, in the last decades a quick growth in synthetic polymers use has been observed, and it is predicted that value could multiply by four by the year 2100 as result of growing human population and prosperity, it will be necessary to use 25% of the world’s current oil production just for plastics manufacture [9]. Taking in account this scenario, one valid option to overcome this environmental problem is the use of bio-based polymers from renewable resources.

This review provides an overview of the application of biodegradable polymers from renewable resources in packaging materials. A wide range of different polysaccharides, their properties, and their state of the art in research and commercial fields are described and discussed.

## 2. Biodegradable Polymers

**Figure 1:**
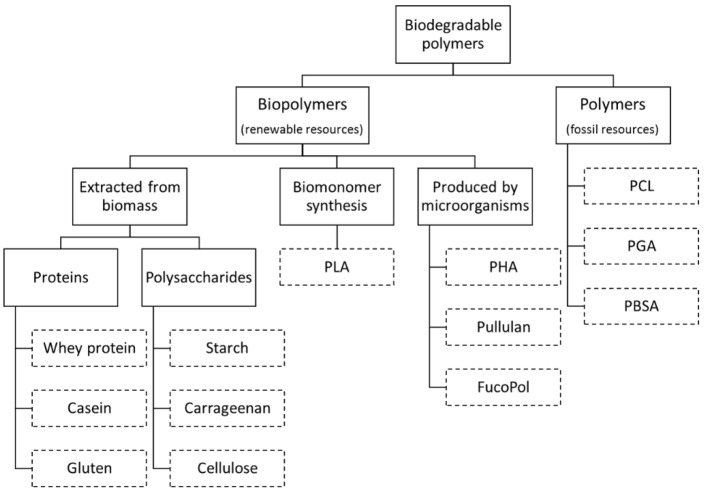
Biodegradable Polymers (Adapted from Encyclopedia of Membranes [18]).

For the polymer industry and consumers it is important to distinguish between biopolymers and biodegradable materials. According to American Society for Testing and Materials (ASTM Standard D-5488-94d [13]), a biodegradable material is defined as “material capable of undergoing decomposition into carbon dioxide, methane, water, inorganic compounds, or biomass in which the predominant mechanism is the enzymatic action of microorganisms, that can be measured by standardized tests, in a specified period of time, reflecting available disposal condition”. On the other hand, biopolymers are polymeric materials derived totally from renewable resources. While biopolymers are biodegradable, not all biodegradable materials are considered biopolymers. As examples, polycaprolactone (PCL), polyglycolide (PGA) and polybutylene succinate adipate (PBSA) are biodegradable materials, but not classified has biopolymers because they are produced from non-renewable resources (fossil-sourced chemicals) (Figure 1) [9].

Biopolymers are generally classified according to their source:

1. Polymers directly extracted/removed from biomass such as polysaccharides (e.g., starch, cellulose, and galactomannans) and proteins (e.g., casein and gluten).
2. Polymers produced by chemical synthesis from renewable bio-derived monomers, such as polylactic acid (PLA), a thermoplastic aliphatic polyester derived from lactic acid monomers. The monomer itself is produced via fermentation of carbohydrate feedstocks.
3. Polymers produced by microorganisms, like some polysaccharides (e.g., gellan gum and pullulan) and polyhydroxyalkanoates (PHA) [14,15].

According to the European Bioplastics organization, biopolymers from renewable resources have to be biodegradable and especially compostable, which allows disposal of the package in the soil, being more energy efficient than recycling, so they can act as fertilizers and soil conditioners [14,16].

Sustainability requires a fine balance between environmental, economic and social concerns. Biopolymers can be considered sustainable in terms of material supply, water and energy use and waste product generation. Moreover, the product viability, human resources and technology development should also be pondered from a point of view of sustainability.

Biodegradable products are usually more expensive than polymers manufactured from petrochemicals, but this circumstance is changing gradually, either by material collection, processing, and conversion technologies, as well as economies of scale. With increasing demands for plastic in the world, consumer concerns about the environment and the use of environmentally friendly products have grown. In addition to that, new regulations have been implemented, namely in EU countries, restricting the use of traditional materials, which led to a great development in biodegradable packaging materials [17].

The idea of using biopolymers (from renewable resources and biodegradable) in packaging, to contribute to sustainable development is recognized, since it is possible to dispose of the plastic waste to be degraded in nature. This solution is particularly interesting for food packaging since these kinds of materials are usually contaminated by food residues that constitute a health hazard in sorting and mechanical recycling [17].

## 3. Polysaccharides in Food Packaging

Polysaccharides are the most abundant macromolecules in the biosphere. These complex carbohydrates constituted by glycosidic bonds are often one of the main structural elements of plants (e.g., cellulose) and animal exoskeletons (e.g., chitin), or have an important role in the plant energy storage (e.g., starch) [19].

A high variety of polysaccharides and their derivatives have been used to produce biodegradable films and thin membranes, and used in several industries, such as food, medical, pharmaceutical and specific industrial processes (e.g., pervaporation) [20]. Polysaccharide-based membranes have been widely used in food industry in packaging and edible coatings. Polysaccharide membranes are generally attractive due to their good barrier against oxygen and carbon dioxide (at low or moderate relative humidity) and good mechanical properties. However, their major drawback is related to their low barrier against water vapor due to their hydrophilic nature [14,21]. The improvement of polysaccharide films has been studied in order to reach satisfactory biopolymer based packaging behavior, possible to use in industrial applications [21]. In the following Section 3.1, Section 3.2, Section 3.3 and Section 3.4, the attention will be focused on polysaccharides application in food packaging.

### 3.1. Polysaccharides Obtained from Animals

#### Chitin and Chitosan

Chitin is the second most abundant agro-polymer produced in nature. It appears naturally in the exoskeleton of arthropods and in the cell walls of fungi and yeasts. It is an acetylated polysaccharide composed of N-acetyl-d-glucosamine and is produced commercially by chemical extraction processes from prawns and crabs wastes. Chitin can also be produced using enzyme hydrolysis or fermentation process, but these processes are not economically feasible yet on industrial scale [19,22].

Chitosan is obtained from deacetylation of chitin, and different factors (e.g., alkali concentration, incubation time, ratio chitin to alkali, temperature and chitin source) can affect its properties. Chitosan is usually insoluble in water, but may be easily dissolved in acidic solutions. Its distinct characteristics from other polysaccharides rely on its cationic groups along the backbone and its antimicrobial properties against bacteria, yeasts and fungi [19,22,23,24]. The good film-forming properties allow the production of membranes (thickness > 30 µm) and coatings (<30 µm) to act as food preservative. Chitosan membranes are biodegradable, biocompatible, non-toxic, renewable and commercially available. Furthermore, chitosan membranes are reported as being semipermeable to gases presenting low oxygen permeability, essential for some food products preservation, and moderate water vapor barrier [23,25,26,27].

Despite these unique properties of chitosan membranes, much research has been done focused on their improvement. Adding glycerol to chitosan membranes, and applying thermo-mechanical treatment (mechanical kneading), it is possible to obtain a kind of thermoplastic material that grants good mechanical properties [19,28].

The functional properties of chitosan-based membranes may also be improved by combination with other hydrocolloids. Blends of chitosan and anionic polymers have been reported to have improved mechanical and barrier properties when compared to those made of chitosan only. This fact is attributed to the formation of polyelectrolyte complexes through electrostatic interactions between the protonated amino groups of chitosan and the negatively charged side-chain groups in the other biopolymer at the operating pH [26,29]. Improvements in mechanical properties, better performance in terms of water vapor permeability and lower water solubility have been reported for combinations of chitosan with other polysaccharides, such as starch, pectin or alginate [29,30,31] and proteins, like gelatin [32] and whey proteins [33], compared to chitosan membranes.

Lipids are usually added to films/membranes to impart hydrophobicity and thereby reduce moisture transfer. A wide range of lipid components is available, such as natural waxes, resins, fatty acids and vegetables oils [34]. A decrease in water susceptibility has been reported for chitosan-based membranes with beeswax [35], and decrease in water vapor permeability was described for chitosan-based membranes with oleic acid [36], neem-oil [37], cinnamon essential oil [38], among others.

The manufacturers and suppliers of chitosan and chitin products are present worldwide. Primex (Siglufjordur, Iceland) commercializes ChitoClear^®^, chitosan products that intend to be based on the purest chitosan possible with potential application in food packaging [39]. Norwegian Chitosan (Kløfta, Norway) trades chitin and chitosan under brand names NorLife and Kitoflokk™, respectively, for several applications, including food and beverages [40]. G.T.C. Bio Corporation (Qingdao, China) which is a chitin and chitosan manufacturer, commercializes different grades of both products with a price around 20 €/Kg for chitin and between 18 and 45 € for chitosan (depending on required purity grade) [41].

### 3.2. Polysaccharides Obtained from Plants

#### 3.2.1. Starch

Starch is the most abundant reserve polysaccharide in plants. As such, it is a renewable resource, biodegradable, produced in abundance at low cost, easy-to-handle and can exhibit thermoplastic behavior. Starch can be extracted from cereals (e.g., corn, wheat or rice), from tubers (e.g., potato, tapioca or manioc), from grain (e.g., amaranth) or even from nuts (e.g., cashew), but commercially, the main sources of starch are corn, potato and tapioca [19,22].

Starch granules are insoluble in cold water and are composed of two types of glucose polymers: amylose (the linear polymer which comprises approximately 20% w/w of starch granules) and amylopectin (the branched polymer). Starch properties depend directly on the botanical source, granule size distribution and morphology, genotype, amylose/amylopectin ratio and other factors such as composition, pH, and chemical modifications [17,22].

This polysaccharide has the ability to form membranes and coatings with very low oxygen permeability, however its applicability as packaging material is dependent on its high hydrophilic character, limited mechanical properties and the retrogradation (increase in crystallinity over time, leading to increased brittleness) [42,43]. Research has been carried out to overcome these drawbacks, mainly using plasticizers, which increase the chain mobility and improve the flexibility, to create starch plastics with mechanical properties comparable to polyolefin-derived ones. The most used plasticizers are polyols such as glycerol, glycol and sorbitol [44,45,46,47].

Other studied approaches consist on designing blends and composites, as well as starch chemical modification to produce a biodegradable material with appropriate mechanical strength, flexibility and water barrier properties for use as packaging material. Blending starch with more hydrophobic polymers is widely studied (e.g., polycaprolactone (PCL) or polylactic acid (PLA)) [48,49], as well as their composites with clay nanoparticles [44,50].

The producers and traders of plastics based on starch include Novamont (Novara, Italy), which commercializes Mater-Bi^®^, a biodegradable and compostable bioplastic commercialized in granular form that can be processed using the most common transformation techniques for plastics [51]. In addition, Eco-Go (Bangkok, Thailand) sells finished packaging products (e.g., bowls, food containers and food trays) from cassava and corn starch [52], and Plantic Technologies Limited (Altona, Australia) produces PLANTIC^TM^, a high barrier multilayer sheet for packaging goods, constituted by corn starch and polyethylene (PE) and polypropylene (PP) [53].

#### 3.2.2. Galactomannans

Galactomannans are neutral polysaccharides obtained from the endosperm of dicotyledonous seeds of several plants, particularly the *Leguminosae*, where they function as carbohydrate reserves [54].

These gums are heterogeneous polysaccharides composed by a β-(1-4)-d-mannan backbone with a single d-galactose branch linked α-(1–6), they differ from each other by the mannose/galactose (M/G) ratio. The three major galactomannans with interest in food and non-food industries are guar gum (*Cyamopsis tetragonolobo*, M/G ratio: 2:1), tara gum (*Caesalpinia spinosa*, M/G ratio: 3:1) and locust bean gum (*Ceratonia siliqua*, M/G ratio: 3.5:1) [54,55]. However, just locust bean gum and guar gum are considered commercially interesting due their availability and price [56].

These natural polysaccharides are commonly used in the food industry, mainly as stabilizers, thickeners and emulsion stabilizers, as well as for the production of edible membranes and coatings. The galactomannans ability to form very viscous solutions at relatively low concentration and their resistance to pH alterations, ionic strength and heat processing are their main distinct characteristics. The mechanical and barrier properties of galactomannan membranes and coatings are the basis of their application to improve the shelf-life, safety and quality of food products [14,54].

Several studies have shown the membrane-forming properties of different galactomannans, being the mannose/galactose ratio, the degree of substitution and the degree of polymerization, the main parameters affecting edible membranes and coatings properties [57].

Edible membranes and coatings of galactomannans have been applied for example in fruit and cheese. They have been tested in apples to decrease the internal oxygen concentration. Sensory analyses revealed that the coated apples maintained consistent quality in firmness, crispness and juiciness [58]. Coatings based on galactomannan, glycerol and corn oil have been applied in cheese, decreasing the transfer rates (water vapor and oxygen), weight loss and color change [59].

Cargill (Minneapolis, MN, USA) offers various types of locust bean gum and guar gum flour or extracts under the trade name Viscogum™. Chemtotal (Chatswood, Australia) also produces and trades galactomannans (guar gum, locust bean gum, tara gum and cassia gum). Other companies producing and commercializing galactomannans include Altrafine Gums (Ahmedabad, India), with exportation to 90 countries of a wide range of different gums.

#### 3.2.3. Cellulose

Cellulose is the most abundant occurring natural polymer on earth, being the predominant constituent in cell walls of all plants. Cellulose is composed of a unique monomer: glucose under its β-d-glucopyranose form [60]. Due to its regular structure and array of hydroxyl groups, it tends to form strong hydrogen bonded crystalline microfibrils and fibers and is most familiar in the form of paper, paperboard and corrugated paperboard in the packaging context [17,22].

Its great interest is related with specific properties such as low density, high mechanical strength, low cost, durability, non-toxicity, renewability, biocompatibility, biodegradability, good films-forming performance, chemical stability and ease of making chemical derivatives [60,61].

The most used raw material source for production of cellulose based products are wood and cotton fibers and in small amounts stalks of sugarcane bagasse. Natural cellulose fibers are low cost, biodegradable and have good mechanical properties, but they are difficult to use for industrial applications due to their hydrophilic nature, insolubility in water and crystallinity [17,19].

Cellulosic materials are usually used in textiles, fibers and packaging and can be divided into two groups: regenerated and modified cellulose. Chemical reactions are usually performed to improve the thermoplastic behavior of cellulose, such as etherification and esterification that are conducted on the free hydroxyl groups. Numerous derivatives are commercialized, but the main ones used for industrial purpose are cellulose acetate, cellulose esters (for extrusion and molding, and, in structures, as membranes) and regenerated cellulose for fibers. To overcome the hard mechanical properties of cellulose, beyond chemical modification, the use of plasticizers and blends with other polymers are also used, being the final mechanical and chemical properties dependent on the blend composition.

To produce cellophane membranes, for example, cellulose has to be dissolved in aggressive and toxic solutions, and then recast in sulfuric acid. In that way, it is possible to produce a hydrophilic layer with good mechanical properties. However, this structure does not have thermoplastic properties and cannot be heat-sealed [17].

Nowadays, a large number of companies are suppliers of cellulose membranes. Innovia Films (Wigton, UK) presents two different products based on cellulose, Cellophane™ and NatureFlex™, which are biodegradable and compostable, both sold worldwide for food packaging applications (Pre-made bags, tapes, box overwrap, bunch wrap, among others) [62]. Weifang Henglian Films CO. LTD (Weifang, China) provides food grade cellulose films with different sizes adapted for specific products.

### 3.3. Polysaccharides Obtained from Algae

#### 3.3.1. Carrageenan

Carrageenan is a naturally occurring hydrophilic, anionic sulfated linear polysaccharide extracted from red seaweeds, specifically from the *Rhodophyceae* family (e.g., *Chondrus crispus, Kappaphycus spp.*, *Eucheuma spp.*, and *Gigartina stellata)* [63,64]. This hydrocolloid is composed of α-d-1,3 and β-d-1,4 galactose residues that are sulfated at up to 40% of the total weight. Carrageenans are classified based on their solubility in potassium chloride, into different types (λ, κ, ι, ε and μ), all composed of 22%–35% sulfate groups, although these designations do not reflect definitive chemical structures [65]. κ-carrageenan is the one with the fewest negative charges per disaccharide having excellent properties to form gels and membranes. When compared with λ- and ι-carrageenan, κ-carrageenan membranes exhibit better mechanical properties [65,66,67].

Carrageenan is approved as food-grade additive, and it has been used mainly as emulsifier and stabilizer in flavored milks, dairy products, pet food, dietetic formulas and infant formulas [65,67].

Carrageenan is also used to produce edible films and coatings, though the reports about its application in coatings are much more common. Carrageenan edible films and coatings and their blends with other polymers were reported to be used in food to preserve fresh cut fruits, by reducing moisture loss and decreasing gas exchange, as well as preventing the discoloration and maintaining texture [68,69]. Membranes of carrageenan have also been reported as encapsulating matrices of aroma compounds [64,70,71].

FMC (Philadelphia, PA, USA) is the largest and the most experienced producer of carrageenan extracts worldwide. Its film-forming carrageenans have brand names of Gelcarin^®^ and Viscarin^®^ [72]. Other important companies in the carrageenan market are CP Kelco (Atlanta, GA, USA), Danisco (Copenhagen, Denmark), Ceamsa (Porriño, Spain), and Quest International (Naarden, The Netherlands). JetNet Corporation (Sewickley, PA, USA) produces carrageenan membranes, in particular Nutrafilm^TM^ carrageenan film packaging for meat and poultry, and over 300 different styles and sizes of elastic netting [73].

#### 3.3.2. Alginate

Alginate is a linear polysaccharide that is abundant in nature and is synthesized by brown seaweeds (e.g. *Laminaria digitata* and *Ascophyllum nodosum*) and some soil bacteria. It has an anionic character and is water-soluble, consisting of monomeric units of 1-4-linked α-d-mannuronate (M blocks) and β-l-guluronate (G blocks), as well as segments of alternating mannuronic and glucuronic acids (MG blocks). The physical properties of alginates depend on the relative proportion of these three blocks, which are directly related with extraction source [74]. They are appealing film-forming compounds because of their non-toxicity, biodegradability, biocompatibility and low cost. In addition, other functional properties have been studied, such as thickening, stabilizing, suspending, gel-producing, among others [22,64,75].

Sodium alginate is the most used in industry and was the first by-product from algal purification. Having an efficient brown seaweed extraction would be interesting for producing an environmentally friendly biopolymer-rich extract for industrial applications, such as food packaging material, release agents, paper, pharmaceutical and medical uses, among others [64]. Due to the linear structure of alginate, the membranes are strong, with adequate fibrous structures in solid state, being considered a good filmogenic material [76].

The market of alginate producers is concentrated in few companies, including FMC (Philadelphia, PA, USA), Cargill (Minneapolis, MN, USA) and DuPont (Danisco) (Copenhagen, Denmark). The price of alginate increased between 2009 and 2013 due to the stronger demand, but stabilized in 2014 at 11 €/Kg [77].

The properties and applications in food packaging of the polysaccharides obtained from animals, plants and algae are summarized in Table 1.

### 3.4. Polysaccharides Obtained from Microorganisms

Several polysaccharides with film-forming ability can be produced by microorganisms (yeast, fungus or bacteria), such as pullulan, gellan gum, xanthan gum, FucoPol, bacterial cellulose or bacterial alginates. This section will focus on the most used polymers except bacterial cellulose and alginate referred before.

#### 3.4.1. Pullulan

Pullulan is a linear, water-soluble and neutral exopolysaccharide (EPS), constituted mainly of maltotriose units connected by α-1,6 glycosidic units and produced by yeast like fungus *Aureobasidium pullulans* using a variety of feedstocks containing simple sugars [22]. The molecular weight of pullulan, ranging from 4.5 × 10^4^ to 6 × 10^5^ Da, is greatly affected by cultivation parameters (temperature, pH, type of carbon source and type of nitrogen source). The commercial production of pullulan began in 1976 by the Hayashibara Company (Okayama, Japan). Its production was an outgrowth of starch syrup production, noted in 1883. Pullulan membranes started to be commercialized by Hayashibara in 1982 [82,83].

Pullulan is biodegradable, non-toxic, tasteless and odorless. It can be used as food additive, as flocculent agent or even as blood plasma substitute, beyond film forming agent. Pullulan membranes are edible, homogeneous, transparent, printable, heat sealable, flexible and good barriers to oxygen [20,84,85]. However, they are water sensitive and mechanically weak [86,87]. These properties, and the fact of pullulan membranes inhibit fungal growth, make them a good material for food applications.

Despite all advantages of pullulan, its high cost has limited the use of pullulan and pullulan membranes in several applications. Research has been carried out on blending pullulan with other biopolymers and additives to produce membranes with better physicochemical characteristics and mechanical properties. Blends of pullulan with alginate, chitosan, cellulose, and starch have been reported with improvements in thermal and mechanical properties, low water vapor permeability and low water absorption [88,89,90,91,92]. Composite membranes of pullulan with lipids and proteins have also shown improved properties. Pullulan membranes with gelatin have demonstrated higher tensile strength and reduced oxygen permeability and cost [93], while the use of rice wax has shown improvements in water vapor barrier properties [86].

Nowadays, apart from the Hayashibara corporation, Shandong Jinmei Biotechnology Co. Ltd. (Zhucheng, China) is also a key producer of pullulan (Jinmei Pullulan), which is commercialized in powder or capsules forms, with application in edible and oral dissolving membranes, coatings in soft candies, among others [94].

#### 3.4.2. Gellan Gum

Gellan gum is an anionic water-soluble exopolysaccharide, produced by *Sphingomonas elodea*, also known as *Auromonas elodea* or *Pseudomonas elodea*. This heteropolysaccharide is a linear high molecular weight (around 5 × 10^5^ Da) compound, with a tetrasaccharide repeating sequence which consists of two residues of β-d-glucose, one of β-d-glucuronic acid and one of α-l-rhamnose [95]. The approximate composition comprises glucose (60%), rhamnose (20%) and glucuronic acid (20%) [96]. Gellan gum was identified as a product with potential commercial value by Kelco (Atlanta, Georgia, USA) during an extensive screening program of soil and water bacteria. In its original form (high acyl gellan), gellan gum has two acyl substituents (acetate and glycerate). Low acyl gellan gum is obtained with removal of acyl groups [95,97]. High acyl gellan forms soft, elastic, non-brittle, thermo-reversible gels, and low acyl gellan tends to form firm, non-elastic brittle and thermostable gels [97,98].

In food industry, gellan gum is usually used as additive (stabilizer, thickening agent and gelling agent), however the applications of gellan gum may also be extended to membranes and coatings for food industry, such as breading and batters for chicken, fish, cheese, vegetables and potatoes, coatings and adhesion systems. These membranes and coatings offer advantages, essentially due to their ability to reduce oil absorption by providing an effective barrier. In batters, for example, product crispness is maintained long after frying or baking, which helps to maintain product quality under heating lamps [99].

CP Kelco (Atlanta, GA, USA) is the leading global producer of gellan gum, commercializing Gelrite™ (low acyl) and Kelcogel™ (high acyl). Dancheng Caixin Sugar Industry co. Ltd (Zhoukou, China) is also a producer and worldwide seller of high and low acyl gellan.

#### 3.4.3. Xanthan Gum

Xanthan gum is an exopolysaccharide produced by *Xanthomonas campestris* using glucose and sucrose as sole carbon source. It was discovered in 1963 at the Northern Regional Research Laboratories (Peoria, IL, USA) and was the second microbial polysaccharide commercialized. Nowadays, it is the most extensively studied and widely accepted industrial microbial biopolymer, being the most significant bacterial EPS in global hydrocolloids market [100,101]. This heteropolysaccharide consists of repeated pentasaccharide units composed og glucose, mannose and glucuronic acid (2:2:1 ratio) and pyruvate and acetyl substituent groups [102].

Xanthan is water-soluble and non-toxic. It imparts a high viscosity at low concentrations in aqueous media, with a strong shear-thinning behavior. The rheological properties of xanthan solutions are quite stable in a wide range of pH, ionic strength and temperature values [103,104].

Xanthan gum has been used in a wide variety of industrial applications, such as food, cosmetic, pharmaceutical, textile, petroleum production or even slurry explosives. In food industry, it is mainly used as additive (suspending and thickening agent) [100,105]. Thus far, there is not much information about xanthan membranes for food packaging, maybe caused by the current high cost of xanthan production [100]. Nevertheless, xanthan coatings applied to acerola, showed it is an effective system for reducing the weight loss and the respiration process, keeping the color and eventually increasing the shelf-life [106].

The major producers include CP Kelco (Atlanta, GA, USA), Danisco (Copenhagen, Denmark), Merck (Kenilworth, NJ, USA), Sanofi-Elf (Gentilly, France) and Jungbunzlauer (Basel, Switzerland) that commercialize xanthan with different purity grades and trade names.

#### 3.4.4. FucoPol

FucoPol is a high molecular weight exopolysaccharide (2–10 × 10^6^ Da) produced by *Enterobacter* A47 (DSM 23139) using glycerol byproduct from biodiesel industry as carbon source. This biodegradable, anionic and water-soluble heteropolysaccharide is composed by fucose (36%–38% mol), galactose (22%–24% mol), glucose (27%–33% mol), glucuronic acid (9%–10% mol) and acyl groups (acetate, succinate and pyruvate), which account for 12–18 wt % of the FucoPol dry weight [107,108,109].

FucoPol production at lab-scale has shown productivities and yields comparable to other commercial microbial bacterial polysaccharides, such as xanthan and gellan [103]. Although this polysaccharide is not commercially available yet, the scale up of its production is being developed.

FucoPol has demonstrated flocculating and emulsion stabilizing capacity, comparable to commercial polymers [110]. FucoPol has also shown to have a good thickening capacity in various aqueous formulations (with a wide range of pH and ionic strength) [111]. These functional properties make this polymer a good alternative in several applications in the food, pharmaceutical, cosmetic, textile, paper and petroleum industries.

FucoPol has also shown membrane-forming capacity. Its membranes have been reported to be transparent, with brownish tone, ductile behavior, water soluble, with low water vapor barrier properties but high barrier properties to gases (in particular CO_2_ and O_2_). Taking ito account these properties, FucoPol based membranes have good potential to be incorporated as an inner layer in a multilayer packaging material [112]. Moreover, FucoPol and chitosan bilayer membranes have shown enhanced properties when compared to FucoPol stand-alone membranes. They exhibited better gas barrier properties, lower solubility in liquid water, and better mechanical properties. These improved properties could support the use of bilayer films (FucoPol/Chitosan) in food packaging with low moisture content products [113].

The properties and applications in food packaging of the microbial polysaccharides are summarized in Table 2.

## 4. Conclusions and Future Perspectives

In this work, the state of the art on polysaccharide-based membranes use for food packaging applications was revised. Polysaccharides extracted from different origins (animals, plants, and algae) and produced by microorganisms have been described. Intensive academic and industry research is being carried out to find new and improved polymers, production methods, sources and properties, to obtain biopolymers (in particular, polysaccharides) that may replace the conventional synthetic and non-biodegradable ones as packaging materials.

The future trends are related with industrial development, able to produce competitive products in performance and price. A detailed life cycle analysis, taking into account all aspects from production costs (still higher for biopolymers) to direct and indirect waste disposal threat costs, is essential to evaluate the economic value of polysaccharide membranes in comparison to their non-biodegradable counterparts. The improvement of existing polysaccharide membranes, particularly regarding their mechanical properties, resistance to liquid water and permeability to water vapor, is mandatory. The strategies may include the use of additives (such as lipids), blends with different polymers, design of multilayered membranes, use of nanoparticles, and polysaccharides chemical modification. This ambitious challenge is crucial for a more sustainable approach in the production of packaging for food products.

## Figures and Tables

**Table 1:** Properties and food applications of polysaccharide membranes obtained from animals, plants and algae.

Polysaccharide	Composition	Membrane Properties	Main Food Applications	Refs
Chitin	N-acetylglucosamine	Biodegradable Antibacterial and fungistatic properties; Biocompatible and non-toxic Highly transparent	Coffee capsules Food bags Packaging films	[24,78,79,80]
Chitosan	d-glucosamine N-acetyl-d-glucosamine	Biodegradable Biocompatible and non-toxic Antifungal and antibacterial properties; Good mechanical properties Barrier to gases High water vapor permeability Brittle—need to use plasticizer	Edible membranes and coatings (Strawberries, cherries, mango, guava, among others) Packaging membranes for vegetables and fruit	[14,28,66,79,81]
Starch	Glucose	Biodegradable Transparent Odorless and tasteless Retrogradation High elongation and tensile strength	Flexible packaging: Extruded bags Nets for fresh fruit and vegetables Rigid packaging Thermoformed trays and containers for packaging fresh food	[17,66]
Galactomannans	Mannose Galactose	Biodegradable EdibleSemi-permeable barrier to gases	Edible membranes and coatings Fruits Cheese	[14,54]
Cellulose	Glucose	Biodegradable Good mechanical properties Transparent Highly sensitive to water Resistance to fats and oils Need to perform modification, use of plasticizer or polymer blend	Cellophane membranes	[17,66]
Carrageenan	Galactose	Biodegradable Fragile and ductile behavior Usually blended with other polymers	Coatings Fruits Meet Encapsulation of aroma compounds	[64,66]
Alginate	Mannuronic Glucuronic acid	Biodegradable High water vapor permeability Poor water resistance Strong and brittle membranes Cross-link with calcium	Coatings Prevent water loss in fresh cut fruit (apple, papaya, pear and melon) Inhibition of microbial growth (turkey products) Microwaveable food (increase warming efficiency)	[14,64,66]

**Table 2:** Properties and application of microbial polysaccharide membranes in food packaging.

Polysaccharide	Microorganism	Composition	Membrane Properties	Main Food Applications	Refs.
Pullulan	*Aureobasidium pullulans*	Maltotriose (three glucose)	Biodegradable Transparent Edible Oil and grease resistant Heat sealable High water solubility Barrier to oxygen	Coating material Wrapping material Blends with other polymers to improvement of mechanical properties Inner package Seasoning bag of instant noodles Instant coffee	[79,82,93]
Gellan gum	*Sphingomonas elodea*	Glucose Rhamnose Glucuronic acid	Biodegradable Edible Lipid barrier Excellent gas barrier Good tensile strength	Edible Coatings in breading and batters for chicken, fish, cheese, vegetables and potatoes. Encapsulation of flavor and bioactive ingredients	[20,95,114]
Xanthan gum	*Xanthomonas campestris*	Glucose Mannose Glucuronic acid AcetatePyruvate	Biodegradable Edible	Edible coating Meet (Prevent moisture migration during frying) Fruit (Extend shelf-life)	[79,106]
FucoPol	*Enterobacter A47*	Fucose Galactose Glucose Glucuronic acid Acetate Succinate Pyruvate	Biodegradable Transparent High gas barrier Poor water resistance	Possible application as inner layer in multilayer packaging	[112,113]
